# Genetic and Clinical Determinants of Chronic Thromboembolic Pulmonary Hypertension: The Role of PAI-1 Polymorphism

**DOI:** 10.3390/ijms27020758

**Published:** 2026-01-12

**Authors:** Özgür Batum, Merve Ayık Türk, Yelda Varol, Berk Özyılmaz, Alp Eren Akarçay, Nigar Dirican, Sibel Doruk, Sami Deniz

**Affiliations:** 1 Department of Pulmonology, University of Health Sciences Turkiye, Izmir Faculty of Medicine, Izmir City Hospital, Izmir 35540, Turkey; ozgurbat@yahoo.com (Ö.B.); yeldavatansever@hotmail.com (Y.V.); aeakarcay@icloud.com (A.E.A.); sami_deniz@yahoo.com (S.D.); 2 Department of Medical Genetics, University of Health Sciences Turkiye, Izmir Faculty of Medicine, Izmir City Hospital, Izmir 35540, Turkey; drberk@gmail.com; 3 Department of Pulmonology, University of Health Sciences, Izmir City Hospital, Izmir 35540, Turkey; nigardirican@yahoo.com (N.D.); sibeldoruk@yahoo.com (S.D.)

**Keywords:** hypertension, pulmonary, chronic thromboembolic, plasminogen activator inhibitor 1, genetic, thrombophilia

## Abstract

Chronic thromboembolic pulmonary disease (CTEPD) is a severe long-term complication of acute pulmonary thromboembolism (PTE). Its pathogenesis is multifactorial, involving incomplete thrombus resolution, hemodynamic burden, comorbidities, and genetic factors. However, the contribution of inherited thrombophilic mutations to CTEPD development remains controversial. This retrospective cohort study included 204 patients diagnosed with acute PTE at a tertiary referral center between December 2023 and December 2024. Baseline demographic, clinical, laboratory, and echocardiographic data were collected. Genetic analysis assessed Factor II, Factor V Leiden, MTHFR C677T, MTHFR A1298C, Factor XIII V34L, and PAI-1 4G/5G polymorphisms. Patients were followed for at least 12 months for the development of CTEPD, defined according to guideline-based hemodynamic and imaging criteria. During follow-up, 17 patients (8.3%) developed CTEPD. Patients with CTEPD were significantly older and had higher baseline and follow-up systolic pulmonary artery pressure (sPAP) (*p* < 0.001), elevated NT-proBNP and troponin levels (both *p* < 0.001), and more frequent comorbidities, including cardiac and renal disease. Multivariate logistic regression identified comorbid diseases (HR: 0.17, 95% CI: 0.039–0.80, *p* = 0.025) and genetic thrombophilic factors (HR: 0.30, 95% CI: 0.10–0.91, *p* = 0.034) as independent predictors. Among genetic variants, only the PAI-1 4G/5G polymorphism was significantly associated with CTEPD (*p* = 0.001). Our study demonstrates that advanced age, comorbid diseases, elevated cardiac biomarkers, and genetic predisposition are associated with the development of CTEPD after acute PTE, while the PAI-1 4G/5G polymorphism may contribute to CTEPD susceptibility within a multifactorial context.

## 1. Introduction

Pulmonary thromboembolism (PTE) is a major clinical condition associated with acute mortality and substantial long-term morbidity. Despite appropriate anticoagulation, incomplete thrombus resolution occurs in a subset of patients, leading to chronic thromboembolic sequelae. Approximately 2–4% of patients who experience acute PTE develop chronic thromboembolic pulmonary hypertension (CTEPH), a form of pulmonary hypertension caused by persistent organized thrombi obstructing the pulmonary vasculature [1,2].

More recently, the broader concept of chronic thromboembolic pulmonary disease (CTEPD) has been introduced, encompassing patients with chronic thromboembolic changes on imaging but without invasive hemodynamic confirmation by right heart catheterization [3]. While CTEPH is the only potentially curable form of pulmonary hypertension, both CTEPH and CTEPD are associated with impaired functional capacity, right ventricular dysfunction, and increased mortality if not recognized and managed appropriately.

The development of chronic thromboembolic disease is believed to be multifactorial. Proposed mechanisms include the initial thromboembolic burden, impaired thrombus resolution, genetic predisposition, and concomitant comorbidities [4,5]. Most available evidence regarding risk factors derives from CTEPH studies, which have demonstrated associations with splenectomy, ventriculo-atrial shunts, malignancy, non-O blood groups, antiphospholipid antibodies, and chronic inflammatory conditions [6,7,8]. Similarly, recurrent embolism, idiopathic PTE, and large residual perfusion defects after acute PTE have been identified as risk factors for progression to chronic thromboembolic disease [6,7,8].

In addition to clinical and thromboembolic risk factors, inherited and acquired thrombophilic conditions may contribute to the pathogenesis of chronic thromboembolic disease. Deficiencies of antithrombin, protein C, or protein S, as well as Factor V Leiden and prothrombin G20210A mutations, elevated factor VIII levels, and hyperhomocysteinemia, have all been implicated [9]. Furthermore, antiphospholipid antibodies, lupus anticoagulant, and other hemostatic abnormalities have been associated with a higher risk of persistent thromboembolic disease [10]. Although thrombophilic factors are well recognized in the occurrence of acute pulmonary thromboembolism, their contribution to thrombus persistence and the development of chronic thromboembolic disease remains incompletely understood.

Emerging genomic studies also suggest that chronic thromboembolic disease may, at least in part, have a polygenic basis. Variants in genes such as FII, TSPAN15, SLC44A2, and FIV have been linked to susceptibility to CTEPH and may also be relevant for CTEPD [11]. However, these findings are not yet incorporated into routine clinical practice, and further research is warranted to clarify their role in diagnosis and risk stratification.

Against this background, the present study aimed to investigate the relationship between genetic thrombophilic factors and the development of CTEPD in patients with acute PTE. In addition, we assessed the impact of comorbidities, biomarkers, and echocardiographic parameters on long-term outcomes. We hypothesized that specific genetic polymorphisms, particularly those influencing fibrinolysis, may increase susceptibility to CTEPD beyond the contribution of traditional clinical risk factors.

## 2. Results 

A total of 204 patients diagnosed with acute pulmonary thromboembolism (PTE) were included in the study, of whom 17 (8.3%) developed chronic thromboembolic pulmonary disease (CTEPD) during follow-up.

### 2.1. Baseline Demographics and Laboratory Findings

Patients who developed CTEPD were significantly older compared to those without CTEPD (57.1 ± 11.4 vs. 46.3 ± 15.1 years, *p* = 0.001). Among laboratory parameters, troponin and NT-proBNP levels at admission were significantly higher in the CTEPD group (55 [80] vs. 10 [45] pg/mL and 1247 [111] vs. 150 [150] pg/mL, respectively; both *p* = 0.001). At baseline echocardiography, systolic pulmonary artery pressure (sPAP) was markedly elevated in the CTEPD group (45 [15] vs. 25 [8] mmHg, *p* = 0.001), while tricuspid annular plane systolic excursion (TAPSE) values were slightly lower (16 [8] vs. 18 [4] mm, *p* = 0.023). During a 6- to 12-month follow-up, sPAP remained significantly higher among CTEPD patients (40 [13] vs. 20 [5] mmHg, *p* = 0.001). Baseline demographic and clinical characteristics are summarized in Table 1.

### 2.2. Clinical and Comorbidity Characteristics

The presence of any comorbidity was more common among CTEPD patients (*p* = 0.033). Specifically, cardiac diseases (*p* = 0.049), chronic kidney disease (*p* = 0.019), and a history of previous deep vein thrombosis (DVT) (*p* = 0.012) were significantly associated with CTEPD development. A history of prior PTE was also more frequent among CTEPD patients (*p* = 0.049). Genetic thrombophilic factors were detected more often in the CTEPD group (*p* = 0.044).

Regarding risk stratification, simplified Pulmonary Embolism Severity Index (sPESI) scores and 30-day mortality risk classifications were significantly higher among patients who later developed CTEPD (*p* = 0.007 and *p* = 0.001, respectively). Radiological or echocardiographic evidence of right heart dilatation at initial diagnosis was also strongly associated with CTEPD (*p* = 0.001). Detailed comparisons are presented in Table 2.

### 2.3. Predictors of CTEPD

In multivariate logistic regression analysis, the presence of comorbid diseases (OR: 0.17, 95% CI: 0.039–0.80, *p* = 0.025) and genetic thrombophilic factors (OR: 0.30, 95% CI: 0.10–0.91, *p* = 0.034) were identified as independent predictors of CTEPD development (Table 3).

### 2.4. Genetic Thrombophilic Factors

The distribution of genetic thrombophilic mutations is presented in Table 4. Factor V Leiden, MTHFR C677T, MTHFR A1298C, and Factor XIII polymorphisms were not significantly different between groups (*p* = 0.284, *p* = 0.415, *p* = 0.632, and *p* = 0.721, respectively). However, the PAI-1 4G/5G polymorphism showed a marked association with CTEPD. Homozygous and heterozygous PAI-1 variants were significantly more frequent among CTEPD patients compared with non-CTEPD patients (82.3% vs. 41.8%, *p* = 0.001).

## 3. Discussion

In this retrospective cohort study, we evaluated clinical, echocardiographic, and genetic risk factors associated with the development of chronic thromboembolic pulmonary disease (CTEPD) after acute pulmonary thromboembolism (PTE). Among the investigated variables, genetic predisposition—most notably the PAI-1 4G/5G polymorphism—emerged as a particularly strong determinant of CTEPD development, whereas traditional thrombophilic mutations such as Factor V Leiden and MTHFR variants showed no significant association. In addition, advanced age, elevated baseline and follow-up systolic pulmonary artery pressure (sPAP), increased NT-proBNP and troponin levels, comorbid diseases, and history of deep vein thrombosis (DVT) were also linked to higher risk.

The incidence of CTEPD in our study cohort was 8.3%. Although most published data report the incidence of hemodynamically confirmed CTEPH after acute PTE, which ranges between 2% and 9% in prospective and registry-based studies [2,3,6], our findings are broadly comparable. This concordance suggests that patients fulfilling non-invasive criteria for CTEPD may represent a population overlapping with, and at risk of progressing to, CTEPH. Consistent with ESC/ERS guidelines, our results underscore the importance of long-term follow-up in patients after acute PTE, given the non-negligible risk of developing chronic thromboembolic sequelae [1,12].

The role of genetic predisposition in chronic thromboembolic disease (CTEPD) remains uncertain, since most available data are derived from studies of hemodynamically confirmed CTEPH. Although the PAI-1 4G/5G polymorphism is not recognized as a risk factor for the occurrence of acute pulmonary embolism, one of the most notable findings of our study was its association with the development of CTEPD. In contrast, classical thrombophilic mutations such as Factor V Leiden, MTHFR polymorphisms, and Factor XIII variants were not significantly different between patients with and without CTEPD, which is consistent with earlier reports in CTEPH populations [9,10]. Lang et al. [10] suggested that impaired fibrinolysis may contribute to the development of CTEPD. The 4G allele of the PAI-1 gene has been associated with increased PAI-1 expression and activity, leading to excessive inhibition of plasminogen activation and reduced fibrinolytic capacity. This mechanism provides a biological link between genetic predisposition and delayed thrombus resolution in CTEPD. PAI-1 is a key inhibitor of fibrinolysis, and elevated PAI-1 levels may therefore promote persistent organized thrombi and progressive vascular remodeling [13]. Recent genomic analyses by Liley et al. [11] further highlight both shared and distinct molecular pathways between acute PTE and chronic thromboembolic sequelae, particularly implicating genes involved in fibrinolysis and vascular remodeling. This observation supports the notion that fibrinolysis-regulating genes may be more relevant to the pathogenesis of CTEPD than classical coagulation-related variants. Although the majority of patients who developed CTEPD carried the PAI-1 mutation, a considerable number of patients without CTEPD also had this genetic variant. Therefore, the predictive value of the PAI-1 4G/5G polymorphism should be interpreted with caution. We acknowledge that while this association was not independent in the multivariate model, its biological plausibility remains noteworthy due to the role of PAI-1 in modulating fibrinolysis and thrombus resolution. Consistent with prior studies, elevated PAI-1 activity or carriage of the 4G allele may impair clot degradation and contribute to persistent vascular obstruction. Thus, the observed relationship likely reflects a contributory rather than determinative effect of this mutation on CTEPD pathogenesis. Furthermore, recent genomic studies underscore the polygenic nature of chronic thromboembolic disease, implicating multiple loci associated with thrombus resolution and vascular remodeling [11,14].

In addition to PAI-1, thrombin-activatable fibrinolytic inhibitor (TAFI) has been identified as another key modulator of fibrinolysis. TAFI attenuates fibrinolysis by removing C-terminal lysine residues from partially degraded fibrin, thereby reducing plasminogen binding and plasmin generation. Several studies have suggested that TAFI gene polymorphisms and elevated TAFI activity may be associated with venous thromboembolism and impaired thrombus resolution. However, TAFI polymorphisms were not assessed in the present study due to the retrospective design and limitations of the predefined genetic testing panel. Future studies incorporating a broader spectrum of fibrinolysis-related genes, including TAFI, may provide a more comprehensive understanding of the genetic determinants of chronic thromboembolic pulmonary disease [15,16]. A recent single-center observational study by Bereczky et al. [17] used next-generation sequencing to interrogate ISTH-recommended Tier 1 and Tier 2 genes related to coagulation, fibrinolysis, and platelet disorders, along with vascular genes, in patients with CTEPH compared with PE controls without CTEPH. The authors reported no single recurrent causative variant, but rather a heterogeneous distribution of rare, non-synonymous variants across multiple pathways, including coagulation, altered fibrinolysis, and impaired angiogenesis, supporting a polygenic disease architecture. Notably, although CPB2 (encoding TAFI) has been proposed as a candidate gene in chronic thromboembolic disease, no exclusive variants in CPB2 were identified among CTEPH cases, underscoring the complexity of fibrinolysis-related genetic contributions and the need for larger, harmonized cohorts. These findings are consistent with our results, which also suggest that genetic factors contribute to CTEPD development within a broader multifactorial framework. In our cohort, the observed association between genetic predisposition and CTEPD supports the concept that chronic thromboembolic disease arises from the interaction of multiple genetic and clinical determinants rather than a single causative variant, highlighting the importance of multifactorial mechanisms in CTEPD pathogenesis.

Beyond the genetic predisposition observed in our cohort, the clinical course of CTEPD may also be influenced by baseline cardiopulmonary status. Given that 17 of 17 patients who developed CTEPD carried the PAI-1 mutation, while a substantial number of mutation carriers did not progress to CTEPD, it is plausible that pre-existing right ventricular function may have played a more prominent role in disease evolution. In our cohort, patients who developed CTEPD had significantly higher NT-proBNP levels and lower TAPSE values at baseline, suggesting subclinical right ventricular strain even at the time of acute PTE. This raises the possibility that impaired baseline cardiac reserve—rather than the PAI-1 genotype alone—may predispose certain individuals to incomplete thrombus resolution and subsequent chronic vascular obstruction. Further analyses exploring the interaction between genetic and functional parameters could help clarify whether the PAI-1 polymorphism exerts its influence primarily through modulation of fibrinolysis or indirectly via exacerbation of right ventricular dysfunction.

Our data demonstrate that elevated baseline and follow-up systolic pulmonary artery pressure (sPAP), increased NT-proBNP and troponin levels, and the presence of right ventricular dilatation were strongly associated with CTEPD. These findings are consistent with prior reports in CTEPH cohorts, where persistent pulmonary hypertension and right heart dysfunction were identified as important markers of adverse outcome [3,4,18,19]. Although invasive hemodynamic confirmation was not performed in our study, the observed echocardiographic and biomarker abnormalities suggest that similar pathophysiological mechanisms may underlie CTEPD. Biomarker elevation, particularly NT-proBNP, has been proposed as a surrogate marker of right ventricular strain and adverse remodeling in both acute and chronic pulmonary vascular disease [20]. Importantly, the elevation of cardiac troponin observed in patients who developed CTEPD may not solely represent acute myocardial injury during the index pulmonary embolism event. Emerging evidence indicates that troponin elevation can also reflect chronic cardiovascular stress, endothelial dysfunction, or subclinical myocardial strain even in the absence of acute ischemia [21].

This concept aligns with our findings, as patients who later developed CTEPD also exhibited higher NT-proBNP levels and echocardiographic signs of right ventricular overload. Therefore, persistent or recurrent troponin elevation in this context may indicate sustained hemodynamic burden and progressive right ventricular remodeling, rather than transient ischemic injury alone.

Comorbid conditions were significantly more common in patients who developed CTEPD. In particular, cardiac disease, chronic kidney disease, and a history of DVT were associated with a higher risk. These findings are in line with prior literature in CTEPH cohorts, suggesting that systemic vascular dysfunction and impaired endothelial repair mechanisms may predispose to chronic vascular obstruction [6,7,22]. Recurrent or unrecognized venous thromboembolism has also been identified as a key risk factor for the development of chronic thromboembolic disease [7,23]. In our cohort, clinical risk stratification tools such as the simplified Pulmonary Embolism Severity Index (sPESI) and early mortality risk scores were significantly higher among patients who later developed CTEPD. These results extend previous findings by indicating that these indices, while originally validated for predicting early mortality in acute PTE, may also be useful for identifying patients at risk of long-term thromboembolic sequelae [24,25,26].

Our findings suggest that patients with elevated sPAP, right ventricular dilatation, comorbidities, or the PAI-1 polymorphism should be considered at higher risk for CTEPD after acute PTE. Routine echocardiographic and biomarker assessment at diagnosis and follow-up may therefore improve early detection. Moreover, in selected patients, particularly those with recurrent thrombosis or unexplained CTEPD, genetic testing could provide additional insights. Identifying patients at risk is crucial, as individuals with CTEPD not only experience significant morbidity but also represent a population at risk of progression to chronic thromboembolic pulmonary hypertension (CTEPH), which is the only potentially curable form of pulmonary hypertension when treated with pulmonary endarterectomy or balloon pulmonary angioplasty [3,5].

In addition to its pathophysiological and genetic implications, it is important to emphasize the clinical significance of chronic thromboembolic pulmonary disease (CTEPD). Although hemodynamic confirmation of pulmonary hypertension was not performed in our cohort, patients with CTEPD frequently experience persistent dyspnea, impaired exercise capacity, and reduced quality of life, representing a condition with substantial morbidity. Recent cohort data indicate that CTEPD (without concurrent pulmonary hypertension) develops in approximately 5–6% of acute PTE survivors, a figure comparable to CTEPH incidence, underscoring its clinical relevance [27]. Moreover, CTEPD represents a heterogeneous clinical spectrum that may reflect an intermediate hemodynamic status and warrants close follow-up, as some cases may evolve toward CTEPH [28,29]. Therefore, identifying predictors of CTEPD is clinically valuable not only for understanding mechanisms of persistent vascular obstruction but also for recognizing patients who may later progress to CTEPH.

The strengths of our study include the systematic assessment of both clinical and genetic factors and the use of multivariate regression analysis. However, several limitations should be acknowledged. First, its retrospective and single-center design may limit the generalizability of our findings. Second, the relatively small number of CTEPD events may have limited the statistical power to detect associations with less prevalent genetic variants. Extending the study period would likely increase event accrual, while a multicenter design could provide a larger and more diverse patient population, allowing more robust analyses of genetic subgroups. Future prospective multicenter studies with longer follow-up and standardized genetic assessment are warranted to validate and extend our findings. Third, although echocardiography was performed according to international standards, invasive right heart catheterization was not systematically available, which precluded definitive confirmation of CTEPH. Therefore, our outcome was defined as CTEPD, and the results should be interpreted within this framework. Finally, only selected thrombophilic mutations were analyzed; future studies incorporating broader genomic approaches, including next-generation sequencing of genes involved in coagulation, fibrinolysis, platelet function, and vascular remodeling, may provide additional insights into the genetic predisposition to chronic thromboembolic disease [17].

In conclusion, our study demonstrates that advanced age, comorbid diseases, elevated cardiac biomarkers, and particularly the presence of the PAI-1 4G/5G polymorphism are significant predictors of chronic thromboembolic pulmonary disease (CTEPD) after acute PTE. These findings underscore the multifactorial pathogenesis of CTEPD, where thromboembolic burden, systemic comorbidities, and genetic predisposition interact. Importantly, the identification of the PAI-1 polymorphism may have practical clinical implications: in patients carrying this mutation, closer surveillance and structured follow-up programs should be considered to facilitate earlier recognition of persistent thromboembolic disease. While CTEPD itself represents a condition associated with substantial morbidity, it also identifies a population at risk of progression to chronic thromboembolic pulmonary hypertension (CTEPH), the only potentially curable form of pulmonary hypertension. Larger prospective studies incorporating comprehensive genomic profiling and invasive hemodynamic confirmation are warranted to validate these associations and to guide personalized risk stratification and follow-up strategies.

## 4. Materials and Methods

### 4.1. Study Design and Population

This retrospective cohort study included patients diagnosed with acute pulmonary thromboembolism (PTE) at a tertiary referral center between December 2023 and December 2024. A total of 204 patients were enrolled. The diagnosis of acute PTE was confirmed using computed tomography pulmonary angiography (CTPA) or ventilation/perfusion (V/Q) scintigraphy, in accordance with current ESC guidelines [1].

Inclusion Criteria

Patients aged ≥ 18 years who were diagnosed with acute pulmonary thromboembolism (PTE) between December 2023 and December 2024 were eligible for inclusion. The diagnosis of acute PTE was confirmed by computed tomography pulmonary angiography (CTPA) or ventilation/perfusion (V/Q) scintigraphy, consistent with the 2019 ESC Guidelines. Only patients who had available baseline demographic, laboratory, echocardiographic, and genetic data and completed at least 12 months of clinical follow-up were included.

Exclusion criteria

(1) Pre-existing pulmonary hypertension of any etiology (Group 1–5 PH according to ESC/ERS classification);
(2) Previously documented chronic thromboembolic pulmonary hypertension (CTEPH) or chronic thromboembolic pulmonary disease (CTEPD);
(3) Acute pulmonary embolism associated with active malignancy under current chemotherapy or radiotherapy;
(4) Severe left-sided heart disease or significant valvular abnormalities on echocardiography;
(5) Chronic lung diseases causing secondary pulmonary hypertension (e.g., COPD GOLD stage ≥ III, interstitial lung disease with FVC < 60%);
(6) Incomplete clinical, echocardiographic, or genetic data;
(7) Lack of adequate follow-up imaging or echocardiographic evaluation after 3 months of anticoagulation.

These criteria ensured the inclusion of patients with newly diagnosed, hemodynamically relevant acute PTE and sufficient follow-up to evaluate the development of CTEPD while minimizing potential confounding conditions.

### 4.2. Data Collection

Demographic and clinical characteristics were recorded, including age, sex, body mass index (BMI), smoking status, and comorbidities such as hypertension, diabetes mellitus, coronary artery disease, malignancy, and chronic obstructive pulmonary disease. The presence of comorbid disease was recorded as a binary variable (yes/no) and defined as the coexistence of at least one chronic condition, including hypertension, diabetes mellitus, cardiac disease, chronic kidney disease, cerebrovascular disease, malignancy, or chronic pulmonary disease. Patients could have more than one comorbidity. History of prior venous thromboembolism (VTE) or PTE, as well as the presence of provoking risk factors such as recent surgery, immobilization, pregnancy, or hormone therapy, were also documented. Hemodynamic and functional parameters were evaluated by transthoracic echocardiography at the time of PTE diagnosis, with particular attention to systolic pulmonary artery pressure (sPAP) and the presence of right ventricular dysfunction. Treatment-related data included the type of anticoagulant therapy administered and the use of systemic thrombolysis.

Blood samples were obtained within 24 h of admission. Routine biochemical analyses, including brain natriuretic peptide (BNP) and high-sensitivity cardiac troponin, were performed in the hospital’s central biochemistry laboratory using standardized automated immunoassay techniques. All measurements were carried out in accordance with the manufacturer’s instructions as part of routine clinical practice, and the results were retrieved from the institutional electronic medical record system. D-dimer levels were measured at baseline for diagnostic confirmation but were not used in follow-up evaluation.

Transthoracic echocardiography was performed in all patients during hospitalization and at follow-up by experienced cardiologists who were blinded to clinical data. Transthoracic echocardiographic examinations were conducted using a GE Vivid S60 ultrasound system (GE Healthcare, Chicago, IL, USA) equipped with a 3Sc-D phased-array transducer. All echocardiographic assessments were performed by experienced cardiologists in accordance with the 2019 ESC Guidelines for the Diagnosis and Management of Acute Pulmonary Embolism [1]. Systolic pulmonary artery pressure (sPAP) was estimated from the tricuspid regurgitant jet velocity using the modified Bernoulli equation (RVSP = 4·V^2^ + estimated right atrial pressure), where right atrial pressure was derived from inferior vena cava (IVC) diameter and its respiratory variation, in accordance with the ASE guidelines [5,6]. Modified Bernoulli equation (RVSP = 4·V^2^ + estimated right atrial pressure), with right atrial pressure derived from inferior vena cava diameter and collapsibility; in the absence of right ventricular outflow obstruction, RVSP was considered equivalent to sPAP [28,29]. Right ventricular (RV) size, function, and the presence of RV dilatation were also evaluated. The following echocardiographic parameters were systematically evaluated: right ventricular (RV) enlargement in the parasternal long-axis view; RV/left ventricular (LV) basal diameter ratio; interventricular septal flattening (“D-shaped” left ventricle); inferior vena cava (IVC) dilatation with reduced respiratory variability; the 60/60 sign (RV outflow tract velocity < 60 cm/s and tricuspid regurgitation gradient < 60 mmHg); tricuspid annular plane systolic excursion (TAPSE); and tricuspid annular systolic velocity (S′). These measurements were used to evaluate RV size, systolic function, and pressure load as part of the standardized echocardiographic protocol applied to all study participants.

Early mortality risk was assessed using the simplified Pulmonary Embolism Severity Index (sPESI; 1 point each for age > 80 years, cancer, chronic cardiopulmonary disease, heart rate ≥ 110 bpm, systolic blood pressure < 100 mmHg, and oxygen saturation < 90%; score 0 designates low risk). Patients were then categorized into four risk groups according to 30-day mortality risk: low risk, intermediate-low risk, intermediate-high risk, and high risk, in line with guideline-based recommendations [1,30,31].

### 4.3. Genetic Analysis

Peripheral venous blood samples were obtained from all patients at the time of acute PTE diagnosis. DNA was isolated from EDTA-anticoagulated samples using standard extraction protocols. Genotyping was performed using the geneMAP Thrombophilia Panel (Genmark, Istanbul, Turkiye), which employs a TaqMan probe-based allelic discrimination system on the LightCycler 480 platform (Roche, South San Francisco, CA, USA).

The PCR reactions included specific primers and fluorescently labeled allele-specific probes for each target polymorphism (Factor V Leiden, Factor II, MTHFR C677T, MTHFR A1298C, Factor XIII V34L, and PAI-1 4G/5G). Amplification was carried out according to the manufacturer’s instructions. The acquired fluorescence data were analyzed using the LightCycler 480 Software (version 1.5.1), and genotypes were assigned automatically based on TaqMan probe-based allelic discrimination curves. Positive and negative controls were included in each run to ensure genotyping accuracy and quality control.

### 4.4. Follow-Up and Outcome Definition

All patients were followed for a minimum of 12 months after the index diagnosis of acute pulmonary thromboembolism.

In this study, right heart catheterization was not systematically performed. Therefore, the primary outcome was defined as chronic thromboembolic pulmonary disease (CTEPD) rather than hemodynamically confirmed CTEPH.

CTEPD was defined as the presence of persistent mismatched perfusion defects on imaging ≥ 3 months after effective anticoagulation, and echocardiographic evidence suggestive of pulmonary hypertension (elevated systolic pulmonary artery pressure and/or right ventricular dysfunction) [12,32].

This pragmatic definition was chosen to reflect clinically relevant chronic thromboembolic disease in the absence of invasive hemodynamic confirmation.

### 4.5. Statistical Analysis

All statistical analyses were performed using SPSS version 23.0 (IBM Corp., Armonk, NY, USA). An a priori sample size estimation was performed using G*Power software (version 3.1.9.7). Based on an expected between-group difference in a key continuous parameter, the calculated effect size was d = 0.938. With α = 0.05, power = 0.95, and the minimum required sample size was 34. Prior to analysis, the distribution of all continuous variables was evaluated for normality using the Shapiro–Wilk test and by inspection of histograms and Q–Q plots. Variables with a normal distribution were expressed as mean ± standard deviation (SD) and compared using the independent samples *t*-test, whereas non-normally distributed variables were presented as median (interquartile range, IQR) and compared using the Mann–Whitney *U* test. Categorical variables were expressed as counts and percentages and compared using the χ^2^ or Fisher’s exact test, as appropriate. Categorical variables were summarized as frequencies and percentages. Between-group comparisons were made using the independent samples *t*-test or Mann–Whitney U test for continuous variables and the χ^2^ test or Fisher’s exact test for categorical variables, as appropriate. Univariate logistic regression was used to identify potential predictors of CTEPD. Variables with *p* < 0.05 were entered into a multivariate logistic regression model. Results were presented as odds ratios (ORs) with 95% confidence intervals (CIs). A two-sided *p*-value < 0.05 was considered statistically significant.

## Figures and Tables

**Table 1:** Baseline demographic, laboratory, and echocardiographic characteristics of patients with and without CTEPD *.

Characteristic	Total (n = 204)	CTEPD + (n = 17)	CTEPD − (n = 187)	*p* Value
Age (years)	46.3 ± 15.1	57.1 ± 11.4	45.3 ± 15	0.001
Cigarette Packs/year	8.7 ± 11.7	9.1 ± 11.6	8.6 ± 11.7	0.93
D-dimer	2010 [2295]	2630 [2240]	1867 [2100]	0.056
Troponin	12 [45]	55 [80]	10 [45]	0.001
NT-ProBNP	150 [150]	1247 [111]	350 [755]	0.001
Diagnosis SPAP	25 [8]	45 [15]	25 [5]	0.001
Diagnosis TAPSE	18 [4]	16 [8]	18 [4]	0.023
6- or 12-month follow-up ECHO SPAP	20 [5]	40 [13]	20 [5]	0.001

* Continuous variables are presented as mean ± standard deviation (SD) or median [interquartile range, IQR]; categorical variables are presented as n (%). *p* values were calculated using Mann–Whitney U test for continuous variables. Abbreviations: sPAP = systolic pulmonary artery pressure; TAPSE = tricuspid annular plane systolic excursion; CTEPD = chronic thromboembolic pulmonary disease.

**Table 2:** Clinical, comorbidity, risk factor, and treatment-related characteristics of patients with and without CTEPD *.

Characteristic	CTEPD + (n = 17)	CTEPD − (n = 187)	*p* Value
Gender			
Male	9 (52.94)	96 (51.33)	1.00
Smoking History			
Never Smoker	10 (58.82)	92 (49.19)	0.50 **
Active Smoker	5 (29.41)	81 (43.31)	
Quit Smoking	2 (11.76)	14 (7.48)	
Comorbid Disease *	15 (88.2)	113 (60.4)	0.033
Hypertension	7 (41.17)	46 (24.59)	0.11
Diabetes Mellitus	2 (11.76)	27 (14.43)	0.55 **
Cardiac Disease	4 (23.52)	14 (7.48)	0.049 **
Cerebrovascular Event	2 (11.76)	6 (3.20)	0.13 **
Atrial Fibrillation	0 (0)	4 (2.13)	1.00 **
Chronic Kidney Disease	2 (11.76)	1 (0.53)	0.019
History of previous PTE	4 (23.52)	14 (7.48)	0.049 **
History of previous DVT	5 (29.41)	14 (7.48)	0.012 **
PTE diagnosis method			
Thoracic Angiography CT	16 (94.11)	161 (86.09)	0.70 **
V/Q	1 (5.88)	26 (13.90)	
PTE Risk Factors ***	1 (5.88)	50 (26.7)	0.077 **
PTE Risk Factors			
No	16 (94.11)	136 (72.72)	0.48
Immobilization	0 (0)	13 (6.95)	
History of Previous Surgery	1 (5.88)	13(6.95)	
Pregnancy	0 (0)	4 (2.13)	
Using Oral Contraceptives	0 (0)	10 (5.34)	
Cancer	0 (0)	11 (5.88)	
Genetic Factor	12 (70.58)	104 (55.61)	0.044 **
sPESI			
0	6 (35.29)	135 (72.19)	0.007
1	10 (58.82)	40 (21.39)	
2	1(5.88)	10 (5.34)	
3	0 (0)	2 (1.06)	
30-day mortality risk score			
High	2 (11.76)	6 (3.20)	0.001
Moderate–high	15 (88.23)	19 (10.16)	
Moderate–low	0 (0)	59 (31.55)	
Low	0 (0)	103 (55.08)	
Thrombolytic Therapy	3 (17.64)	11 (5.88)	0.098 **
Treatment			
DMAH	0 (0)	22 (11.76)	0.43
Warfarin	8 (47.05)	65 (34.75)	
OAK	9 (52.94)	100 (53.47)	
Right dilatation present at initial diagnosis (on CT/ECHO)	12 (70.58)	5 (2.67)	0.001 **

Clinical, comorbidity, and risk factor characteristics of patients with and without CTEPD assessed at the time of initial diagnosis of acute pulmonary thromboembolism (baseline). Values are presented as n (%) for categorical variables. Values are presented as n (%), where percentages are calculated within each subgroup (CTEPD + or CTEPD −). Comparisons between groups were performed using the chi-square or Fisher’s exact test for categorical variables. * Comorbid disease indicates the presence of one or more of the listed chronic conditions. Some patients had multiple comorbidities. ** Fisher’s exact test was used for variables with expected cell counts < 5. *** “PTE risk factors” indicates the presence (yes/no) of at least one known provoking condition for acute pulmonary thromboembolism. The subsequent row lists the specific types of risk factors (immobilization, recent surgery, pregnancy, oral contraceptive use, or malignancy) among those with a positive history. Abbreviations: sPESI: simplified Pulmonary Embolism Severity Index, CTEPD = chronic thromboembolic pulmonary disease, PTE: Pulmonary Thromboembolism, DVT: Deep Vein Thrombosis.

**Table 3:** Logistic regression analysis for predictors of CTEPD *.

	OR (95% CI)	*p* Value
History of PTE (yes/no)	0.37 (0.10–1.37)	0.14
Comorbid Diseases (yes/no)	0.17 (0.039–0.80)	0.025
Genetic Factors (yes/no) *	0.30 (0.10–0.91)	0.034

The logistic regression model was adjusted for history of pulmonary thromboembolism (PTE), presence of comorbid diseases, and genetic thrombophilic factors. Values are presented as odds ratios (OR) with 95% confidence intervals (CI). * For clarity, the variable was recoded so that 1 = absence and 0 = presence of a genetic factor. In this configuration, OR = 2.72 (95% CI 0.91–8.15; *p* = 0.073), indicating that genetic predisposition increases the odds of developing CTEPD. Abbreviations: PTE: Pulmonary Thromboembolism, OR: Odds Ratio, CI: Confidence Interval, CTEPD = chronic thromboembolic pulmonary disease.

**Table 4:** Distribution of genetic thrombophilic factors in patients with and without CTEPD.

	CTEPD + (n = 17)	CTEPD − (n = 187)	*p* Value
Factor II			
Normal	13 (76.5)	172 (91.9)	0.07
Heterozygous	4 (23.5)	15 (8.1)	
Homozygous	0 (0)	0 (0)	
Factor V Leiden			
Normal	12 (70.5)	151 (80.7)	0.46
Heterozygous	5 (29.5)	32 (17.1)	
Homozygous	0 (0)	4 (2.2)	
MTHFR C677T			
Normal	10 (58.8)	91 (48.6)	0.26
Heterozygous	7 (41.2)	83 (44.4)	
Homozygous	0 (0)	13 (7)	
MTHFR A1298C			
Normal	8 (47.0)	73 (39)	0.21
Heterozygous	9 (53)	88 (47)	
Homozygous	0 (0)	26 (14)	
PAI-1			
Normal	0 (0)	52 (27.8)	0.001
Heterozygous	5 (29.4)	91 (48.6)	
Homozygous	12 (70.6)	44 (23.6)	
Factor XIII (V34L)			
Normal	11 (64.7)	132 (70.5)	0.93
Heterozygous	5 (29.4)	47 (25.2)	
Homozygous	1 (5.9)	8 (4.3)	

Values are presented as n (%). Percentages were calculated within each subgroup (CTEPD + or CTEPD −). Comparisons between groups were performed using the chi-square or Fisher’s exact test, as appropriate. Abbreviations: CTEPD = chronic thromboembolic pulmonary disease.

## Data Availability

The data that support the findings of this study are available from the corresponding author upon reasonable request. Due to institutional regulations, the dataset is not publicly archived.
